# Patients’ and professionals’ perspectives on implementation of opportunistic salpingectomy: a mixed-method study

**DOI:** 10.1186/s12913-021-06767-9

**Published:** 2021-07-25

**Authors:** Malou E. Gelderblom, Laura A. M. Van Lieshout, Jurgen M. J. Piek, Joanne A. De Hullu, Rosella P. M. G. Hermens

**Affiliations:** 1 Department of Obstetrics and Gynecology, Radboud Institute for Health Sciences, Radboud University Medical Centre, PO Box 9101, 6500 HB Nijmegen, The Netherlands; 2 Department of Obstetrics and gynecology, Catharina Cancer Institute, Catharina Hospital, Eindhoven, The Netherlands; 3 Department of IQ Health Care, Radboud Institute for Health Sciences, Radboud University Medical Centre, Nijmegen, The Netherlands

**Keywords:** Salpingectomy, Primary prevention, Ovarian neoplasms, Risk reduction, Implementation science

## Abstract

**Background:**

To prevent ovarian cancer, several international societies have issued guidelines which recommend to discuss opportunistic salpingectomy with women undergoing pelvic surgery after completion of childbearing. The opportunistic salpingectomy refers to the additional removal of Fallopian tubes during pelvic surgery for another indication to reduce the risk of developing ovarian cancer. These recommendations emphasize the importance of counselling on benefits and risks of opportunistic salpingectomy but offer no guidance on their implementation in daily practice. The lack of a tailored implementation strategy has resulted in a wide variation in current practice. To reduce this practice variation, we identified influencing factors on implementing opportunistic salpingectomy from patients’ and professionals’ perspectives.

**Methods:**

We conducted a mixed-method study between 2019 and 2020 throughout the Netherlands. In a qualitative phase, we conducted interviews with gynecologic patients (*N* = 11) and their professionals (*N* = 20) to explore barriers and facilitators, using an interview guide. In the quantitative phase, we quantified these barriers and facilitators among patients who underwent a hysterectomy or sterilization and were counselled on the opportunistic salpingectomy (*N* = 77), and members of the Dutch Society of Obstetrics and Gynecology (*N* = 204), using questionnaires. For both phases, barriers and facilitators were classified into the following domains: innovation, patient, healthcare professional, social setting, organization, and economic and political context.

**Results:**

For patients, main barriers were lack of knowledge about: the existence of the opportunistic salpingectomy (45%), size of the surgery (44%) and its associated possible disadvantages (37%). In addition, patients attributed their reluctance to concerns about the removal of healthy organs (46%). For professionals, main barriers were: patients’ lack of knowledge of the size of surgery (85%) and its associated possible disadvantages (77%), the gap in evidence on long term risks and benefits (43%), the lack of feasibility in certain patients and during vaginal surgery (66%). Both patients (41%) and professionals (67%) identified the need for counselling material as facilitator.

**Conclusion:**

To reduce the variety in care regarding opportunistic salpingectomy, consensus and uniform counselling is needed. Including the opportunistic salpingectomy in gynecological guidelines and a decision aid for counselling could serve as tools to facilitate implementation.

**Supplementary Information:**

The online version contains supplementary material available at 10.1186/s12913-021-06767-9.

## Introduction

Ovarian cancer is the main cause of death among patients diagnosed with a gynecological malignancy. The vast majority of ovarian malignancies are epithelial ovarian cancers (EOC), which have a poor five-year survival rate [1]. This poor prognosis results from diagnosis in an advanced stage of disease, often due to late onset of symptoms and lack of early detection methods. Furthermore, comprehensive treatment swiftly results in recurrent disease, for which curative treatment options are limited.

Interest in primary prevention of ovarian cancer has increased following the identification of the Fallopian tube as the main origin for the most common subtype of EOC: high-grade serous ovarian carcinomas (HGSOC). HGSOCs are thought to develop from Serous Tubal Intra-Epithelial Carcinomas in Fallopian tube epithelium [2–4]. Removal of the Fallopian tubes might therefore lower HGSOC incidence, as shown in large cohort studies where ovarian cancer incidence was reduced following salpingectomy [5, 6]. The lifetime risk for ovarian cancer of approximately 1.3% in the general population does not warrant elective preventive surgery in itself, as the risks of general anesthesia and surgery outweigh the ovarian cancer risk [1]. However, ovarian cancer risk could be reduced through opportunistic salpingectomy (OS): the additional removal of Fallopian tubes during pelvic surgery for another indication [5–8].

Although cohort studies show benefits, results from randomized controlled trials regarding long term outcomes are not expected for many years. Given the opportunistic nature of OS, several international societies have issued guidelines which recommend to discuss OS with all women undergoing abdominal surgery after completion of childbearing [9]. These recommendations emphasize the importance of counselling on benefits and risks of OS but offer no guidance on their implementation in daily practice, which results in a wide variation in current practice among hospitals and individual gynecologists [10]. This practice variation, often based on the professionals’ preference instead of the patients’, is undesirable.

To reduce practice variation, optimal implementation of new recommendations concerning OS is essential. Adaptation of daily practice is difficult and requires an implementation strategy tailored to the needs of patients and healthcare professionals, which is currently lacking. According to the model of Change of Grol & Wensing, the first step in the development of a tailored strategy is to gain insight into factors influencing implementation of OS from stakeholders’ perspectives [11]. In this study, we aim to identify the barriers and facilitators in implementing OS from both patients’ and professionals’ perspectives.

## Material and methods

### Study design and integration

**Fig. 1:**
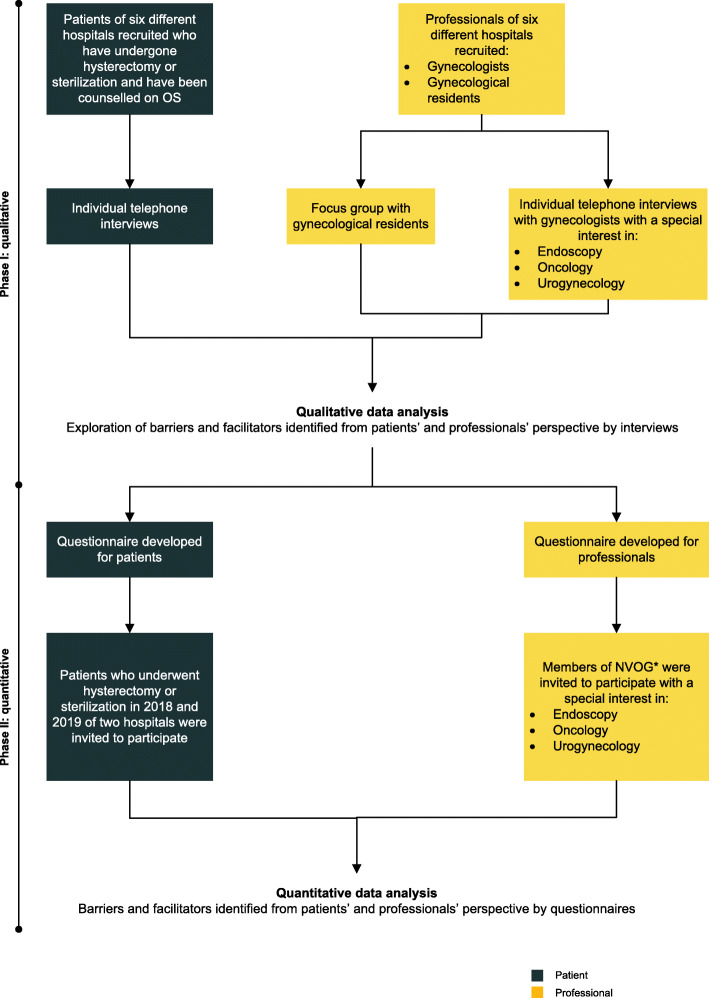
Flowchart of study design. Footnote ^*^ the Dutch society for Obstetrics and Gynecology (NVOG) consists predominantly of gynecologists, but contains some gynecological residents as well

We conducted a mixed-method study using the two-phase Exploratory Sequential Design with a qualitative following a quantitative phase [12]. In the qualitative phase using a qualitative descriptive design [13], we explored possible barriers and facilitators for the implementation of OS through individual in-depth telephone interviews with patients and professionals, and one focus group with gynecological residents. To assess the importance in daily practice of the barriers and facilitators found, cross-sectional questionnaires [14] were developed for patients and professionals to quantify the identified barriers and facilitators. For integration of the data, we first analyzed the qualitative data for identifying barriers and facilitators and then used these findings to develop questionnaires for measuring its importance (building) [12]**.** Figure 1 provides an overview of the study design. This study was described following the Good Reporting of a MM Study (GRAMMS) [15], the consolidated criteria for reporting qualitative research (COREQ) [16] (Additional file 1) and the Consensus-Based Checklist for Reporting of Survey Studies (CROSS) [17] (Additional file 2).

### Qualitative phase

#### Patients

Patients were eligible for participation if they underwent hysterectomy or sterilization in 2018 or 2019 and were counselled on OS. Patients were purposively sampled and approached for individual telephone interviews (to lower the threshold for participation) by their treating gynecologists from six hospitals (one university hospital and five large teaching hospitals) throughout the Netherlands to account for regional differences.

#### Professionals

Gynecologists with a special interest in endoscopy, oncology or urogynecology were approached for individual telephone interviews by one researcher (LvL). Gynecologists approached via the network of the Dutch Society of Obstetrics and Gynecology (NVOG) were asked to participate or suggest a colleague for participation. Professionals were approached by the researcher taking into account dispersion throughout the Netherlands. Solely gynecologists specialized in gynecological surgery were recruited as they were expected to be most exposed to OS. The gynecologists were employed in 12 hospitals (one university, nine teaching and two non-teaching) throughout the Netherlands to account for regional differences. In addition, gynecological residents of the same hospitals were given the opportunity to represent the perspective of future gynecologists in a focus group that was guided by a chairman (LvL). The participating residents represented different years of training.

#### Data collection

Separate semi-structured interview-guides were developed in advance by one researcher (LvL) based on literature and experience within the research team, and tested among two patients and two professionals. An expert in qualitative research (RH) and two gynecologists (JP, JdH) evaluated and approved the interview-guides. The two interviewers (MG, LvL) were medical doctors, had experience in conducting qualitative research and had no relationships with participants prior to the study. Patients and professionals were questioned on their knowledge and personal experience in the decision and counselling of OS. To identify barriers and facilitators on different domains classified according to the frameworks of Grol & Wensing [18] and Flottorp [19], patients and professionals were questioned on: innovation (OS), patient, healthcare professional, social context, organization, and economic and political context. Questions were open-ended, with optional questions to deepen each domain. Interviews were conducted after receiving written informed consent and verbal permission for audiotaping the interview. Data collection was considered complete when data saturation was reached and no new barriers and facilitators were identified for two consecutive interviews. All interviews were audiotaped after the interviewer introduced herself, commented on the research team, and described the study purpose, procedure and funding. Introduction of the study and person identifiable data were not audiotaped for privacy reasons. Moreover, field notes were written after each interview.

#### Data analysis

All interviews were transcribed verbatim, and two researchers (MG, LvL) independently coded the transcripts using ATLAS.ti (version 7.5.15, Atlas.ti Scientific Software Development GmbH; Berlin, Germany). Transcripts were not returned to participants for comments or feedback. First, all interviews were fully read and phrases were descriptively labelled by open coding. Second, comparable descriptive codes were combined and redefined into specific subthemes. The subthemes were merged into the six broader domains of Grol & Wensing [18] and Flottorp [19] using axial coding. After each step findings were compared and disagreements were discussed with a third researcher (RH) until consensus was reached. Participants did not provide feedback on the findings.

### Quantitative phase

#### Patients

All patients who underwent hysterectomy or sterilization in 2018 and 2019 in two hospitals (one university hospital and one large teaching hospital), were invited to participate in a questionnaire by their treating gynecologist. Patients were sampled using the purposive sampling technique and represented women who were eligible for OS. Therefore, patients were not selected for age assuming that hysterectomy or sterilization was not performed in case of uncompleted childbearing. Patients were excluded from participation and completion of the questionnaire if OS was not discussed during counselling. Written information was sent on behalf of their gynecology department.

#### Professionals

Members of the NVOG with a special interest in endoscopy, oncology and urogynecology were invited by email to participate in a questionnaire. We used the purposive sampling technique for sampling. Members were predominantly gynecologists, but included gynecological residents as well.

#### Data collection

Based on the results of the interviews (qualitative phase), two researchers (MG, RH) developed separate web-based questionnaires for patients and professionals in the online tool ‘LimeSurvey’ [20]. For convenience, the patient questionnaire was also available on paper. Both questionnaires started with a section on baseline characteristics followed by statements on qualitatively identified barriers and facilitators that could be answered according the 4-point Likert Scale (strongly disagree, disagree, agree, and strongly agree). The option ‘not applicable’ was omitted to elicit an answer. The patient questionnaire contained 28 questions and was pilot tested by seven women, which was then adapted based on their feedback. The professionals questionnaire contained 46 questions and was pilot tested by two gynecologists and five gynecology residents as well. Initially all questions were mandatory but as the questionnaire was cut short if people felt unable to answer, all questions were made optional and could be left blank. Participants received information on the aim of the study and questionnaire. The patient invitation and questionnaire was sent by mail or e-mail if the e-mail address was accessible. Patients who received the invitation by mail had the choice to complete the questionnaire on paper or online via an URL. The questionnaire for professionals was sent by mass mail and accessible via an URL. Completing the questionnaire took approximately 5–10 min. All data was processed anonymously and collected in an electronic database using Castor EDC (Electronic Data Capture) between January 2020 and June 2020.

#### Data analysis

Data was analyzed using SPSS (IBM Corp. Released 2016. IBM SPSS Statistics, version 24.0). Baseline characteristics were descriptively analyzed and presented as percentage or mean value. Descriptive statistics were used to calculate the agreement with the statements. Responses reported were categorized in the scores ‘strongly disagree’, ‘disagree’, ‘agree’ and ‘strongly agree’. In the text, ‘Disagree’ represented all ‘strongly disagree’ and ‘disagree’ responses amalgamated. ‘Agree’ represented all ‘strongly agree’ and ‘agree’ responses amalgamated.

Internal consistency of the questionnaires was assessed with Cronbach’s alpha per (sub) domain to estimate the reliability [21]. All Cronbach’s alfa values were > 0.6, indicating an acceptable level of reliability except for the professionals’ subdomain ‘Patient: beliefs and knowledge’ (Additional file 3).

## Results

An overview of the baseline characteristics of all participants is provided in Table 1. A staged narrative approach is used for reporting qualitative and quantitative findings [12]. The qualitative data is discussed first, an overview is provided in Table 2. Subsequently, the barriers and facilitators most frequently identified in quantitative analysis are discussed by domain.

**Table 1:** Baseline demographics of patients and professionals in interviews and questionnaires

	Interviews	Questionnaires
	**Patients** **(*N*** **= 11)**	**Patients** **(*N*** **= 77)**
Duration interviews in minutes (mean – SD)	19.8 (± 4.2)	–
Age (mean – SD)	42.1 (± 7.2)	43.0 (± 6.5)
Level of education		
Primary / pre-vocational school	0	8 (10%)
Vocational education	3	26 (34%)
Pre-college education / college	1	30 (39%)
University	0	12 (16%)
Missing	7	1 (1%)
Type of surgery		
Hysterectomy	6	59 (77%)
Sterilization	5	18 (23%)
Menopausal state		
Premenopausal	10	60 (78%)
Menopausal	0	14 (18%)
Post-menopausal	1	2 (2%)
Missing	0	2 (2%)
Family history of ovarian cancer		
First-degree relative	1	2 (3%)
Second-degree relative	2	3 (4%)
None	1	70 (91%)
Unknown / missing	7	2 (3%)
Made the decision on whether or not to undergo OS herself		
Yes, patient chose for OS	7	55 (71%)
Yes, patient chose against OS	2	14 (18%)
No, clinician made the decision	2	6 (8%)
Other	0	2 (3%)
Counselled by gynecologist	11	74 (96%)
Missing	0	3 (4%)
Satisfied with the decision		
Yes	9	70 (91%)
No	0	3 (4%)
Missing	2	4 (5%)
	**Professionals** **(*N*** **= 20)**	**Professionals** **(*N*** **= 204)**
Gynecologists	12 (60%)	195 (94%)
Duration interviews in minutes (mean – SD)	24.9 (± 7.3)	–
Age (mean – SD)	47.4 (± 7.3)	46.8 (± 7.8)
Work experiences in years	12 (IQR 7–16)	10 (IQR 5–17)
Gender		
Female	9	132 (68%)*
Male	3	63 (32%)*
Special interest^1^		
Benign and/or endoscopy	2	130 (67%)*
Oncology	11	83 (43%)*
Urogynecology	7	57 (29%)*
Other	0	6 (3%)*
Type of hospital^2^		
Academic	1	40 (21%)*
Teaching hospital	9	110 (57%)*
Non-teaching hospital	2	50 (26%)*
Gynecological residents	8	9 (4%)
Duration interviews in minutes (mean)	40	–
Age (mean – SD)	32.5 (± 2.9)	34.4 (± 1.5)
Gender		
Female	7	7
Male	1	2
Year of residency		
1 or 2	1	0
3 or 4	5	0
5 or 6	2	9
Special interest^1^		
Benign and/or endoscopy	0	7
Oncology	2	2
Urogynecology	6	5
Other	0	0

*Percentage of gynecologist or gynecological residents subgroup; ^1^ Gynecologists and gynecological residents might have multiple special interests; ^2^ Some gynecologists were employed in multiple hospitals

**Table 2:** Barriers and facilitators identified during interviews and focus group by patients and professionals

Domain	Barriers	Facilitators
**Identified by patients**		
Innovation (OS)	• Low life time risk of ovarian cancer in general population • Insufficient evidence of long-term risks and effects	• Reduction of ovarian cancer risk • Family history of ovarian cancer • Fallopian tubes lose function after completion of childbearing • High lethality rate of ovarian cancer
Patient	• Unwillingness to have healthy organs removed • Lack of insight into the size of surgery • Worry if OS fails • Complicated choice whether or not to undergo OS	• Reliable information material such as a decision aid • Counselled and advised by their gynecologists • Confidence in treating physician • A small additional scar in case of sterilization is not a problem
**Identified by professionals**		
Innovation (OS)	• Low life time risk of ovarian cancer in general population • Presence of residual risk of ovarian cancer after OS • Risk of overtreatment • Insufficient evidence of long-term risks and effects • Complicating the surgery, especially in patients with certain medical history • More difficult during vaginal surgery • More extensive surgery as sterilization method • Unclear limits of the eligible population	• Reduction of ovarian cancer risk • High lethality rate of ovarian cancer • High success rate for OS • No increase in complication risk compared to complication risk of the primary procedure • No extension of surgery in case of a hysterectomy • Fallopian tubes lose function after completion of childbearing • Family history of ovarian cancer
Health care professional	• Unaware or not convinced of evidence • Insufficient skills to perform OS • Experiencing time pressure during consultation due to counselling for OS • Forgetting to counsel about OS	• Uniform counselling material such as a decision aid • Performing a national prospective follow up study for OS registration and ovarian cancer
Patient	• Unwillingness to have healthy organs removed • Unwillingness to take unnecessary risks • Fear of earlier menopause • Lack of knowledge concerning the disadvantages • Lack of insight into the size of surgery • Lack of knowledge concerning the difference between ovaria and fallopian tubes • Complicated choice	• High acceptance among patients • High awareness of OS • Not worrying if performing OS fails
Organization	• Limited time to provide counselling • Increased surgical time, especially for sterilization • More time and pathologists needed for analysis of the Fallopian tubes • Additional (telephone) consultation required	• Counselling for OS possible during regular consultation • No additional surgical instruments are required
Social		• National consensus on OS • Communal policy about OS in gynecological department • Inclusion of OS in the guidelines of the Dutch society for Obstetrics and Gynecology (NVOG) • Inclusion of OS in the guideline of the Dutch College of General Practitioners (NHG) • Inclusion of the recommendation to discuss OS in several guidelines of international societies
Economic and Financial	• Higher costs due to Fallopian tube analysis by pathologists • Higher costs due to increase in surgical time in case of sterilization • Invoicing of OS is unclear	• Cost-effectiveness on long term due to opportunistic nature • No extra costs in case of additional intervention

### Qualitative phase

#### Patients

Thirteen patients were approached for participation in an individual interview. Participation was refused by two patients since they did not respond to invitation. Interviews were conducted with 11 patients between June 2019 and February 2020. Six patients underwent a hysterectomy and five a sterilization. Two patients made the decision against OS. Interviews took 15 to 25 min and were conducted by one of two researchers (MG, LvL).

#### Professionals

All gynecologists who were approached to participate participated. Individual interviews were conducted with 12 gynecologists between December 2019 and March 2019. Individual interviews took 15 to 40 min. Gynecologists had mainly oncology as special interest and their median work experience duration was 12 years. Additionally, eight gynecological residents participated in a focus group interview and took approximately 40 min.

#### Barriers and facilitators

**Fig. 2:**
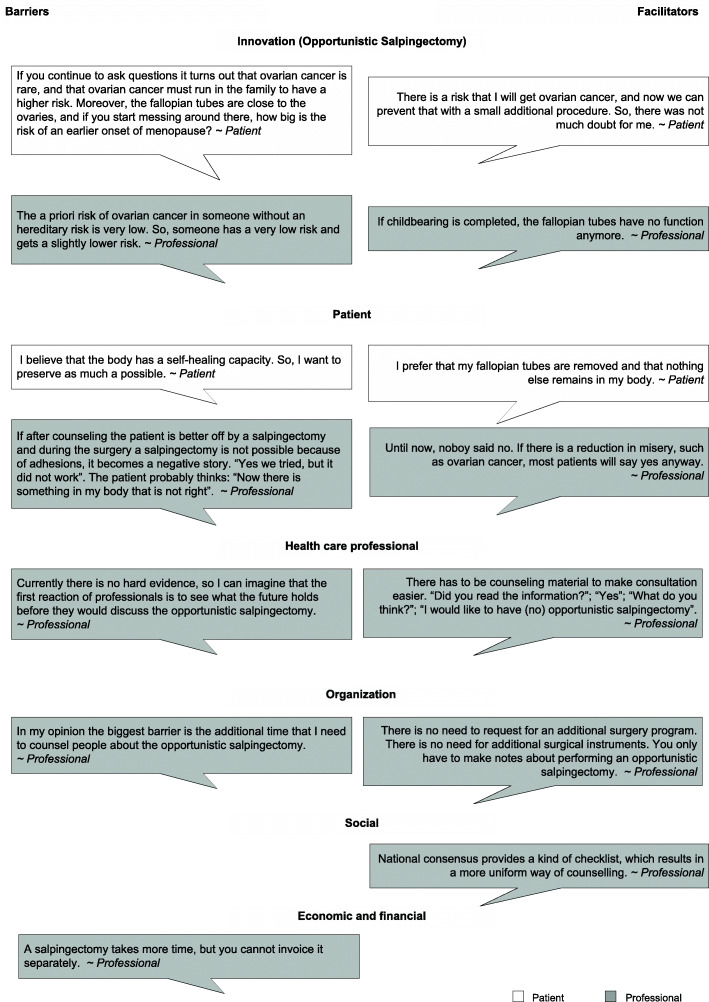
Illustrative quotations from patients and professionals concerning barriers and facilitators on implementation of OS

Patients identified six barriers and eight facilitators in only two domains; ‘innovation’ and ‘patient’. Professionals identified 26 barriers and 21 facilitators in all domains (Table 1), ten of them were identified by both patients and professionals. Figure 2 illustrates barriers and facilitators in implementing OS identified in interviews by domain.

Several barriers and facilitators appeared consistently throughout all interviews. As a result, they were clear based on the qualitative data. We refrained from verification through questionnaires to limit the length of the questionnaire. In the domain innovation this concerned: the low life time risk of ovarian cancer in the general population and the residual risk after OS as barriers, mentioned by both patients and professionals. The reduction of ovarian cancer risk combined with the high lethality of this disease, the loss of tubal function after completion of childbearing, and family history of ovarian cancer were appointed as facilitators.

### Quantitative phase

#### Patients

The questionnaire was sent to 287 patients and yielded a response rate of 37% (*n* = 106); 27 patients were excluded as they were not counselled on OS, and two patients did not complete the questionnaire. Responses of 77 patients were included for analysis. Patients represented different educational levels and had a mean age of 43 years (SD 6.5). Furthermore, 69 patients made the decision for or against OS themselves, 55 of which choose for OS.

#### Professionals

The questionnaire was sent to approximately 600 members of the NVOG with special interest in endoscopy, oncology and urogynecology and yielded a response rate of 34%. All 204 responses were included for analysis: 94% of the respondents were gynecologists, 56% was employed in a teaching hospital. Both gynecologists and residents had mainly benign and/or endoscopy as special interest. The median work experience duration among gynecologists was 10 years (IQR 5–17).

#### Barriers and facilitators

**Fig. 3:**
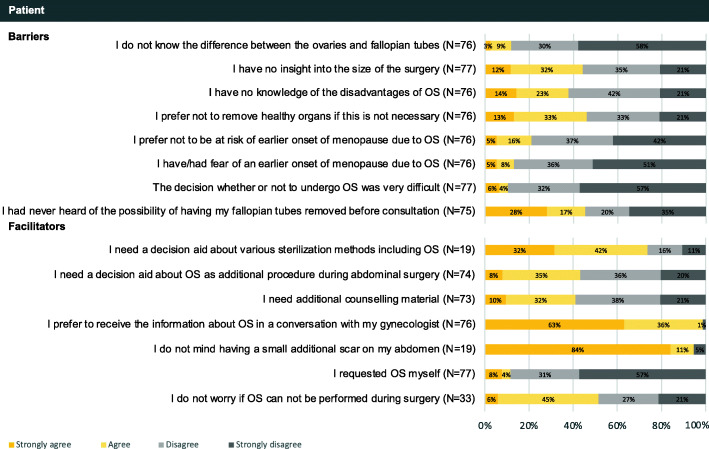
Patients’ responses to statements regarding barriers and facilitator towards implementation of OS by domain

**Fig. 4:**
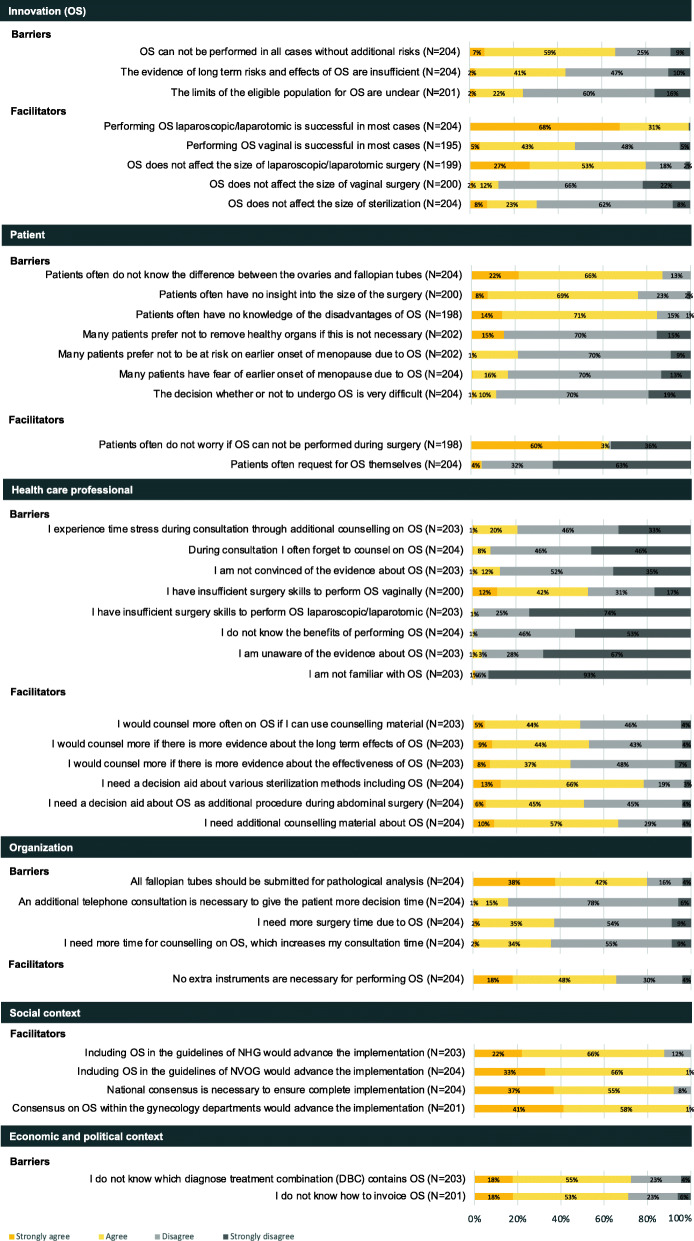
Professionals’ responses to statements regarding barriers and facilitator towards implementation of OS by domain

The patients’ questionnaire contained eight barrier and nine facilitator statements exclusively in the subdomain ‘patient’ (Fig. 3). The professionals’ questionnaire contained 24 barrier and 18 facilitator statements in all domains (Fig. 4).

##### Innovation (OS)

Professionals indicated that OS cannot be performed in all cases without additional risks due to adhesions and vaginal approach (66%), and the insufficient evidence on the long-term effects such as age at onset of menopause (43%) as barriers. Almost all professionals (99%) mentioned the high success rate of OS as facilitator for the laparoscopic/laparotomic approach.

##### Patient

Patients mentioned three barriers related to patients’ knowledge: 1. lack of knowledge on the possible disadvantages of OS (37%); 2. no insight in the size of the surgery (44%), in which size refers to the potential complications and health impact associated; 3. and never heard of the possibility of OS prior to consultation (45%). The first two barriers were identified as barrier by professionals as well (85 and 77% respectively). Regarding patients’ motivation, 46% of patients attributed their reluctance to concerns about removing healthy organs if this was not required. Moreover, patients would be concerned if OS could not be performed during surgery because of an increased risk of complications (52%). In contrast, professionals (63%) indicated that patients were often unconcerned if OS could not be performed.

Regarding facilitators, 40% of the patients mentioned the need for counselling material complementary to consultation with their gynecologist, in particular for a patient decision aid (PtDA) about OS during abdominal surgery (43%) and the various sterilization methods including OS (72%).

##### Health care professional

Professionals mentioned one barrier concerning insufficient skills to perform OS by vaginal approach (53%) and six facilitators related to their needs and preferences such as additional counselling material about OS (67%). A PtDA about OS as addition to abdominal surgery (51%) and the various sterilization methods including OS (78%) could facilitate counselling. Furthermore, professionals indicated they would counsel for OS more often if they had access to counselling material (49%), and if more evidence existed about its effectiveness with regards to risk reduction (45%) and long-term effects of OS such as the onset of menopause (53%).

##### Organizational context

Professionals mentioned the need for more surgery time (38%), and an increase of the consultation duration (36%) as barriers. Of the professionals, 80% reported that all Fallopian tubes should be submitted for pathological analysis.

##### Social context

According to 99% of the professionals, a clear policy on OS within the individual gynecology departments would contribute to implementation of OS. To ensure complete implementation of OS national consensus would be necessary (92%). Including OS in the guidelines of the NVOG (99%) would facilitate the implementation.

##### Economic and political context

Professionals mentioned not knowing how OS should be invoiced (72%) and which diagnosis treatment combination contains OS (72%) as barriers.

## Discussion

In this mixed-method study, we identified influencing factors for the implementation of OS from patients’ and professionals’ perspective through semi-structured interviews and questionnaires. In interviews, patients indicated solely barriers and facilitators in the innovation and patient domain. Professionals indicated barriers and facilitators in all domains. Both stakeholders consistently mentioned the overall low ovarian cancer risk and residual cancer risk after OS as main barriers. Cancer risk reduction and high lethality of ovarian cancer were main facilitators together with the loss of tubal function after completion of childbearing and family history of ovarian cancer. Subsequently, questionnaires determined the importance of barriers and facilitators found. Among other things, both stakeholders indicated patients lack of knowledge about OS as barrier, and the availability of adequate counselling material as facilitator.

From a patients’ perspective, the most important barrier in deciding whether or not to undergo OS is that they are simply unaware of its existence. Patients also lack knowledge on the advantages, disadvantages and the magnitude of the intervention, which prevents a well-informed decision. Adequate information on all aspects of the surgery is essential to enable patient participation in decision making [22]. The content should be based on the patients’ knowledge needs and fully explain all aspects that could potentially influence the decision for patients.

Whether or not patients want to undergo OS depends on their personal beliefs and values. Although healthcare professionals often feel they know what is important for patients, this may not always be true [23]. Our findings show discrepancies between patients’ and professionals’ responses on the domain of patient emotions and motivation, for example on patients’ concerns following a failed OS or that some patients are reluctant about the removal of healthy organs if this is not strictly necessary. Uniform counselling material could counteract the current practice variation caused by professionals’ preferences in patient counselling, especially due to the preference sensitive nature of this decision [24]. Incorporating the counselling (material) of OS in gynecological guidelines subsequently creates consensus [25].

An important barrier for professionals is the gap in evidence on the long-term effects of OS, especially regarding the onset of menopause. OS could potentially accelerate onset of menopause through a reduction of the blood supply to the ovaries and a subsequent diminished ovarian reserve. Earlier menopause could affect sexual wellbeing, quality of life and increased cardiovascular disease risk [26, 27]. Although short-term follow-up studies do not show a decreased ovarian reserve due to OS in women who underwent hysterectomy, a recent review [28] indicates a possible effect consistent with an earlier onset of menopause with a maximum of 20 months. In spite of the probability that clear evidence on long term effects will take several more decades, the popularity of OS is increasing. Therefore, until more evidence is obtained from currently ongoing trials such as HOPPSA (ClinicalTrials.gov;NCT03045965, [29]) and STOPOVCA-young (ClinicalTrials.gov;NCT04757922), it is crucial to inform patients that OS may lead to a slightly earlier onset of menopause.

Professionals questioned the feasibility of OS in patients with adhesions. Since these patients have an increased risk of complications during abdominal surgery, it raises the question whether the benefits continue to outweigh the risks. The same applies to OS during vaginal surgery; professionals indicate they may lack the skills to perform OS via the vaginal route. These findings are likely to be related to the inaccessibility of the Fallopian tubes and less experience with the vaginal approach. However, the vaginal approach should not be a contraindication to OS since professionals report a high success rate [30]. Moreover, a vaginal approach is often preferable as these patients return to normal activities more quickly [31]. Professional should remain aware of the opportunistic and preventive nature of OS and should refrain from performing OS if the benefits do not outweigh the risk. Informing patients of this caveat will provide reassurance if the intention of OS cannot be fulfilled.

The main organizational barrier to implement OS is the additional time it takes in several aspects of daily practice, particularly: 1. additional surgical time due to OS, which reflect the findings of McAlpine et al., 2016 [32], and Jones et al., 2017 [33]; 2. the need for additional counselling time to discuss OS, leading to longer consultations; 3. additional routine examination of the Fallopian tubes requires time of the pathologist. This touches on the barriers found on the financial level, indicating the uncertainties about invoicing of OS, since OS is performed in addition to an already planned surgery. A short-term solution is required through national regulations regarding billing. Although OS may incur additional health care costs in the short-term, long-term health care costs concerning ovarian cancer will decline as OS will reduce the number of cancer cases [34, 35].

Our study shows a variation in current practice regarding OS by the proportion of patients who were not counselled and did not receive any information about OS. This is in contrary with the international guidelines that recommend discussion of OS with all women undergoing gynecological abdominal surgery after completion of childbearing. To reduce this variation, the uptake of OS by professionals might be promoted through exposure of the recommendations from multiple sources [36]. Both our findings and the study by Jones et al., 2017 [33] suggest a source like a guideline outlining the safety, potential benefits and risks of OS. Moreover, the mentioned barriers on lack of knowledge and counselling can be assuaged by the development of a PtDA, according to the criteria of the International Patient Decision Aid Standards Collaboration [37]. In this counselling tool, the decision regarding OS needs to be deliberated on complete information of (potential) disadvantages and benefits. In this way a PtDA about OS could ease both stakeholders needs for counselling material, improve patients’ knowledge, assist counselling by professionals and function as source.

The main strength of our study was that we used mixed-methods to identify possible influencing factors from the perspective of both patients and professionals. The in-depth interviews of phase 1 explored a wide range of detailed barriers and facilitators, while the questionnaires of phase 2 provided insight into whether these barriers and facilitators were more personal or if they were supported by a majority of the group. We used the theoretical frameworks of Grol&Wensing and Flottorp for exploration of both barriers and facilitators influencing implementation of OS. Clearly, there are limitations of the present study. First, the use of these frameworks could be argued since patients indicated influencing factors in solely two domains, while factors that facilitate or hinder implementation in clinical practice might affect various domains. However, our findings suggest that this is not applicable to patients in the implementation of OS. It could conceivably be hypothesized that the minor addition of OS not noticeably affects the care process for patients. Secondly, the inclusion of solely professionals experienced with gynecological surgery and the skewedness towards the special interest oncology might bias the existing barriers and facilitators in the entire gynecological field. For example, obstetricians who are less likely to perform an OS, might have other insights because of their less experience in adnexal surgery. Generalizability of our finding can therefore be questionable. Finally, some statements were identified as both a barrier and a facilitator during the interviews. To prevent participants from feeling that they had to answer the same question twice, we included a statement as a barrier if the statement was more often referred to as a barrier during the interviews and vice versa. However, if this statement was not confirmed as barrier during the quantitative phase it remained unclear if it would have been a facilitator instead.

## Conclusion

We identified barriers and facilitators affecting the implementation of OS. To reduce the variety in care regarding OS, consensus among gynecologists on the indication for OS and the content of uniform counselling seems needed. Gynecological guidelines and a PtDA could serve as implementation tools for both stakeholders.

## Supplementary Information

**Additional file 1.** Explanation of the COREQ checklist

**Additional file 2.** Explanation of the CROSS checklist

**Additional file 3.** Cronbach’s alfa per (sub) domain of patients’ and professionals’ questionnaire. Since we developed our own questionnaires as our research questions were very specific, we calculated the Cronbach’s alpha to assess reliability. All Cronbach’s alfa values were > 0.6, indicating an acceptable level of reliability with the exception of the ‘patient domain’ in the professionals’ questionnaire indicating that these results should be interpreted with caution.

**Additional file 4.** Patients’ questionnaire

**Additional file 5.** Professionals’ questionnaire

## Data Availability

The datasets used and/or analyzed during the current study are available from the corresponding author on reasonable request.

## Abbreviations

EOC: Epithelial ovarian cancer
HGSOC: High-grade serous ovarian carcinomas
NVOG: Dutch society of obstetrics and gynecology
OS: Opportunistic salpingectomy
PtDA: Patient decision aid
