# Supplementary material for: Patients’ and professionals’ perspectives on implementation of opportunistic salpingectomy: a mixed-method study
Source: BMC Health Serv Res. 2021 Jul 25;21:736. doi: 10.1186/s12913-021-06767-9 (PMC8310584; doi:10.1186/s12913-021-06767-9)
Supplement: Supplementary file 1 — Additional file 1. Explanation of the COREQ checklist [file 12913_2021_6767_MOESM1_ESM.docx]

**ADDITIONAL FILE 1** Explanation of the COREQ checklist

| Domain | Comment | Reported on page number or not applicable (N/A) |
| --- | --- | --- |
| Domain 1: Research team and reflexivity | | |
| Personal Characteristics | | |
| Interviewer / facilitator | Individual interviews with professionals were conducted by one researcher (LvL). Individual interviews with patients were conducted by one of two researchers (MG, LvL). A focus group with gynecologic residents was guided by one chairman (LvL). | 9 |
| Credentials | RH has extensive experience in conducting qualitative health research. The other four research team members are medical doctors and skilled in conducting qualitative health research. | 6 |
| Occupation | Both interviewers (MG, LvL) are full time PhD students at the Radboud Institute for Health Sciences at the time of conducting the research. | 1 |
| Gender | The research team consists of one man and four women. | N/A |
| Experience and training | Both interviewers (MG, LvL) have experience in conducting qualitative health research. | 6 |
| Relationship with participants | | |
| Relationship established | Both interviewers had no relationships with participants prior to the study. Therefore, the interviewers should be seen as unbiased. | 6 |
| Participant knowledge of the interviewer | The interviewer introduced herself stating that she was a PhD student, employed at the Radboud Institute for Health Sciences, and commented on the research team, the purpose of the research and its funding. | 6 |
| Interviewer characteristics | Both interviewers were medical doctors and full time PhD students within gynecology. They were aware of recent literature on the topic prior to the start of this research. | 6 |
| Domain 2: Study design | | |
| Theoretical framework | | |
| Methodological orientation and Theory | Barriers and facilitators were identified at the six domains using the frameworks of Grol & Wensing and Flottorp. | 6, 8 |
| Participant selection | | |
| Sampling | Purposive sampling is used. | 5 |
| Method of approach | Participants were recruited by their treating gynaecologists.  Gynecologists with a sub specialism in endoscopy, oncology or urogynecology were approached by mail for individual telephone interviews by one researcher. | 5, 6 |
| Sample size | 12 Gynecologists, 8 Gynecologic residents, 11 Patients | 9 |
| Non-participation | A number of potential participants did not respond to the invitation to participate. However, no person replied with a rejection of participation and everyone who agreed to participate did participate. | 8 |
| Setting | | |
| Setting of data collection | All individual interviews were conducted by telephone to lower the threshold for participation in the research. | 5 |
| Presence of non-participants | None | N/A |
| Description of sample | Description of sample is presented in results section. | 9 |
| Data collection | | |
| Interview guide | Separate semi-structured interview-guides for patients and professionals were developed in advance by one researcher (LvL) based on literature and experience within the research team, and tested among two patients and two professionals. An expert in qualitative research (RH) and two gynecologists (JP, JdH) evaluated and approved the interview-guides. | 6 |
| Repeat interviews | Repeat interviews were not carried out. | N/A |
| Audio / visual recording | All interviews were audiotaped and transcribed verbatim.  Audio recording was turned on after the interviewer’s introduction and participant’s approval for recording. | 7 |
| Field notes | Field notes were written after each interviews regarding the nature of the interview. | 6 |
| Duration | Duration of interviews varied between 14 to 40 minutes. Individual interviews with patients were generally around 20 minutes. Individual interviews with professionals were generally around 23 minutes. | 9 |
| Data saturation | Data collection was considered complete when data saturation was reached and no new barriers and facilitators were identified for two consecutive interviews. | 7 |
| Transcripts returned | Transcripts were not returned to participants for comments and/or corrections. | 7 |
| Domain 3: analysis and findings | | |
| Number of data coders | Two researchers (MG, LvL) independently coded the transcripts. Each interviewer coded the data and described codes. These codes were regularly discussed with the other interviewer. | 8 |
| Description of the coding tree | First all interviews were fully read and phrases were descriptively labelled by open coding. Second, comparable descriptive codes were combined and redefined into specific subthemes. The subthemes were merged into the six broader domains of Grol & Wensing and Flottorp using axial coding and the grounded theory method. | 8 |
| Derivation of themes | The used themes were according to the six domains of the frameworks of Grol & Wensing and Flottorp. | 6, 8 |
| Software | ATLAS.ti (version 7.5.15, Atlas.ti Scientific Software Development GmbH; Berlin, Germany). | 7 |
| Participant checking | Participants did not provide feedback on the findings. | 7 |
| Reporting | | |
| Quotations presented | Quotations are presented in figure 2 and confidentially identified. | Figure 2 |
| Data and findings consistent | There is consistency between the data presented and the findings. | Results section |
| Clarity of major themes | Major themes are clearly identified. | Results section |
| Clarity of minor themes | Minor themes are clearly identified and related to the major themes. | Results section |
