# Supplementary material for: Patients’ and professionals’ perspectives on implementation of opportunistic salpingectomy: a mixed-method study
Source: BMC Health Serv Res. 2021 Jul 25;21:736. doi: 10.1186/s12913-021-06767-9 (PMC8310584; doi:10.1186/s12913-021-06767-9)
Supplement: Supplementary file 2 — Additional file 2. Explanation of the CROSS checklist [file 12913_2021_6767_MOESM2_ESM.docx]

**ADDITIONAL FILE 2** Explanation of the CROSS checklist

| Section/topic | Item | Item description | Reported on page number or not applicable (N/A) |
| --- | --- | --- | --- |
| Title and abstract |  |  |  |
| Title and abstract | 1a | The word “questionnaire” along with a commonly used term was stated in the abstract to introduce the study’s design. | 2 |
|  | 1b | An informative summary in the abstract was provided, covering background, objectives, methods, findings/results, interpretation/discussion, and conclusions. | 2 |
| Introduction |  |  |  |
| Background | 2 | A background about the rationale of study was provided, what has been previously done, and why this survey is needed. | 3 |
| Purpose/aim | 3 | We aimed to identify the barriers and facilitators in implementing OS from both patients’ and professionals’ perspectives. | 3 |
| Methods |  |  |  |
| Study design | 4 | We conducted a mixed-method study using the two-phase Exploratory Sequential Design with a qualitative following a quantitative phase. A cross-sectional study design was used for the quantitative phase. | 5 |
| Data collection methods | 5a | Each questionnaire contained two sections, one about baseline characteristics and one about statements regarding the implementation of the opportunistic salpingectomy. The number of questions were respectively 28 for the patients’ questionnaire, and 46 for the professionals’ questionnaire. | 8 |
|  | 5b | Study population for the quantitative phase is reported in the method section. The 4-point likert scale was used to answer statements on qualitatively identified barriers and facilitators. Internal consistency of the questionnaires was assessed with Cronbach’s alpha. | 8, 9 |
|  | 5c | Both questionnaires were pilot tested once. The patient questionnaire was pilot tested by seven patients. The professional questionnaire was pilot tested as well by two gynecologists and five gynecology residents. All pilot testers had the same inclusion criteria as the study population | 8 |
|  | 5d | The questionnaires are fully provided as additional files. | Additional file 4  Additional file 5 |
| Sample  characteristics | 6a | The study population is described in the method section. | 7, 8 |
|  | 6b | The study population is sampled by using the purposive sampling technique. Women eligible for OS of different Dutch hospitals were recruited. |  |
|  | 6c | The sample size is described in the results section of the manuscript. | 10 |
|  | 6d | Participants represented women who were eligible for OS. | 7 |
| Survey  administration | 7a | The patient questionnaire was sent by mail (on paper) and/or e-mail (web-based) if e-mail address was accessible. Patients who received the invitation by mail had the choice to complete the questionnaire on paper or online via an URL. All data was processed anonymously and collected in an electronic database using Castor EDC. | 8 |
|  | 7b | Data collection took place between January 2020 and June 2020. | 8 |
|  | 7c | Non-web-based surveys were sent by mail. The questionnaire was completed and then returned anonymously to the hospital.  Web-based: The survey was only accessible online via an online URL. When registering online, a unique token was sent to the e-mail address that could be used once. | NA |
| Study preparation | 8 | The questionnaires were developed by two researchers (MG, RH) based on the results of the interviews. Subsequently both questionnaires were pilot-tested to assess usability. Invitations and questionnaires were eventually sent by mail or e-mail to the study population. | 8 |
| Ethical  considerations | 9a | Ethical approval for the survey is obtained including informed consent, institutional review board [IRB] approval, Helsinki declaration, and good clinical practice [GCP] declaration. | 17 |
|  | 9c | All data was processed anonymously. All data of the web-based questionnaire was automatically converted to this anonymous token making it impossible to link the responder to the corresponding survey. All data of the questionnaire on paper were converted to an anonymous record in CastorEDC by a researcher. | 8 |
| Statistical analysis | 10a | Statistical methods, analytical approach and analysis are described in the method section. | 8, 9 |
|  | 10b | Not applicable | NA |
|  | 10c | We performed a complete case analysis. Responders who did not complete the questionnaire were excluded for analysis. Questions that did not apply to a responder or were deliberately left blank, were omitted per statement. Figure 3 and 4 show the number of responses per statement. No other methods were used to deal with missing data. | Figure 3,  figure 4 |
|  | 10d | To avoid non-response errors the sender (the hospital) was clearly stated, confidentiality was guaranteed, a reminder was sent, and the online questionnaire was available on different devices. The number of nonresponses might be a result of refusing to participate in the questionnaire, being incapacitated to answer the questionnaire and by losing the questionnaire. | NA |
|  | 10e | Not applicable | NA |
|  | 10f | Not applicable | NA |
|  | 10g | Not applicable | NA |
| Results |  |  |  |
| Respondent  characteristics | 11a | The numbers of study population are reported in the result section. | 10 |
|  | 11b | Non-participation might be a result from refusing to participate in the questionnaire, being incapacitated to answer the questionnaire and by losing the questionnaire. | NA |
|  | 11c | The response rates were respectively 34% and 37%. The response rate is calculated by the formula: (the number of participants who responded / the number of invited participants) x 100%. | 10 |
|  | 11d | Not applicable | NA |
| Descriptive results | 12 | Characteristics of study participants are provided in the results section, as well as assessed outcomes | 10 |
| Main findings | 13a | Give unadjusted estimates and, if applicable, confounder-adjusted estimates along with 95% confidence intervals and p values. | NA |
|  | 13b | For multivariable analysis, provide information on the model building process, model fit statistics, and model assumptions (as appropriate). | NA |
|  | 13c | Provide details about any sensitivity analysis performed. If there are considerable amount of missing data, report sensitivity analyses comparing the results of complete cases with that of the imputed dataset (if possible). | NA |
| Discussion |  |  |  |
| Limitations | 14 | Limitations of the study are discussed in the discussion section. | 16 |
| Interpretations | 15 | An overall interpretation of results is given in the discussion section. Suggestions are discussed for future research. | 13, 14 |
| Generalizability | 16 | Since the study population does not cover all gynecologist who could counsel for OS, the generalizability of our findings can be questionable. | 16 |
| Other sections |  |  |  |
| Role of the funding  source | 17 | The funding had no role in the survey design, implementation of analysis. | 17 |
| Conflict of interest | 18 | There is no conflict of interest. | 17 |
| Acknowledgements | 19 | Acknowledgements are provided in the acknowledgement section. | 16 |
