# Supplementary material for: Patients’ and professionals’ perspectives on implementation of opportunistic salpingectomy: a mixed-method study
Source: BMC Health Serv Res. 2021 Jul 25;21:736. doi: 10.1186/s12913-021-06767-9 (PMC8310584; doi:10.1186/s12913-021-06767-9)
Supplement: Supplementary file 3 — Additional file 3. Cronbach’s alfa per (sub) domain of patients’ and professionals’ questionnaire. Since we developed our own questionnaires as our research questions were very specific, we calculated the Cronbach’s alpha to assess reliability. All Cronbach’s alfa values were > 0.6, indicating an acceptable level of reliability with the exception of the ‘patient domain’ in the professionals’ questionnaire indicating that these results should be interpreted with caution. [file 12913_2021_6767_MOESM3_ESM.docx]

**ADDITIONAL FILE 3** Cronbach’s alfa per (sub)domain of patients’ and professionals’ questionnaire

| **Domain** | **Number of items** | **Number of respondents** | **Cronbach’s alfa** | **Mean score (SD)** |
| --- | --- | --- | --- | --- |
| **Patients’ questionnaire** | | | | |
| Patient  Emotion/motivation  Beliefs and knowledge  Needs  Preferences | 5  5  2  1 | 33  72  72  . | 0.703  0.746  0.736  . | 0.952 (0.233)  2.148 (2.527)  1.271 (0.000) |
| **Professionals’ questionnaire** | | | | |
| Innovation (OS) | 8 | 187 | 0.630 | 2.660 (0.285) |
| Patient  Emotion/motivation  Beliefs and knowledge | 4  3 | 201  195 | 0.604  0.544 | 2.025 (0.007)  2.962 (0.018) |
| Health care professional  Knowledge and skills  Cognition  Needs and preferences | 6  3  6 | 200  202  201 | 0.728  0.675  0.780 | 1.493 (0.106)  1.764 (0.017)  2.610 (0.026) |
| Social setting | 4 | 200 | 0.727 | 3.276 (0.017) |
| Organizational factors  Recourses | 3 | 204 | 0.641 | 2.266 (0.003) |
| Economic and political context | 2 | 200 | 0.933 | 2.845 (0.001) |

Since we developed our own questionnaires as our research questions were very specific, we calculated the Cronbach’s alpha to assess reliability. All Cronbach’s alfa values were >0.6, indicating an acceptable level of reliability with the exception of the ‘patient domain’ in the professionals’ questionnaire indicating that these results should be interpreted with caution.
