# Supplementary material for: Patients’ and professionals’ perspectives on implementation of opportunistic salpingectomy: a mixed-method study
Source: BMC Health Serv Res. 2021 Jul 25;21:736. doi: 10.1186/s12913-021-06767-9 (PMC8310584; doi:10.1186/s12913-021-06767-9)
Supplement: Supplementary file 4 — Additional file 4. Patients’ questionnaire [file 12913_2021_6767_MOESM4_ESM.docx]

**ADDITIONAL FILE 4** Patients’ questionnaire

Baseline characteristics

1. What is your age?
2. What is your level of education?
   1. Primary / pre-vocational school
   2. Vocational education
   3. Pre-college education / college
   4. University
   5. Other
3. What is your menopausal status?
   1. Premenopausal
   2. Menopausal
   3. Post-menopausal
4. Do you have children?
   1. Yes > How many children do you have?
   2. No
5. Would you (still) like to have children in the future?
   1. Yes
   2. No
   3. I don’t know
6. Does ovarian cancer run in your family?
   1. Yes > Who has ovarian cancer in your family?
   2. No
   3. I don’t know
7. Have you ever had surgery in your abdomen?
   1. Yes > How often have you had abdominal surgery?
   2. No
8. Have you had any gynecological surgery in the past two years?
   1. Yes
   2. No

*If question 8 answered with yes:*

1. What gynecological surgery did you have?
   1. I had a hysterectomy
   2. I had a sterilization
2. Was it discussed with you at that time that it was possible to remove your fallopian tubes?
   1. Yes
   2. No
3. Who informed you about the removal of the fallopian tubes?
   1. My gynecologists
   2. My general practitioner
   3. Other
4. Have you made a decision about whether or not to have your fallopian tubes removed?
   1. Yes, I made the decision myself for removal of my fallopian tubes
   2. Yes, I made the decision myself against removal of my fallopian tubes
   3. No, the clinician made the decision for me
   4. Other, ..
5. Are you satisfied with your decision?
   1. Yes
   2. No
6. Would you briefly explain why?

Your situation

1. I had never heard of the possibility of having my fallopian tubes removed before consultation
   1. Strongly disagree
   2. Disagree
   3. Agree
   4. Strongly agree
2. I requested OS myself
   1. Strongly disagree
   2. Disagree
   3. Agree
   4. Strongly agree
3. The decision whether or not to undergo OS was very difficult
   1. Strongly disagree
   2. Disagree
   3. Agree
   4. Strongly agree
4. I have/had fear of an earlier onset of menopause due to OS
   1. Strongly disagree
   2. Disagree
   3. Agree
   4. Strongly agree
5. I prefer not to be at risk of earlier onset of menopause due to OS
   1. Strongly disagree
   2. Disagree
   3. Agree
   4. Strongly agree
6. I have no knowledge of the disadvantages of OS
   1. Strongly disagree
   2. Disagree
   3. Agree
   4. Strongly agree
7. I have no insight into the size of the surgery
   1. Strongly disagree
   2. Disagree
   3. Agree
   4. Strongly agree
8. I do not worry if OS can not be performed during surgery
   1. Strongly disagree
   2. Disagree
   3. Agree
   4. Strongly agree
9. I do not know the difference between the ovaries and fallopian tubes
   1. Strongly disagree
   2. Disagree
   3. Agree
   4. Strongly agree
10. I do not mind having a small additional scar on my abdomen
    1. Strongly disagree
    2. Disagree
    3. Agree
    4. Strongly agree
11. I prefer to receive the information about OS in a conversation with my gynecologist
    1. Strongly disagree
    2. Disagree
    3. Agree
    4. Strongly agree
12. I need additional counselling material
    1. Strongly disagree
    2. Disagree
    3. Agree
    4. Strongly agree
13. I need a decision aid about OS as additional procedure during abdominal surgery
    1. Strongly disagree
    2. Disagree
    3. Agree
    4. Strongly agree
14. I need a decision aid about various sterilization methods including OS
    1. Strongly disagree
    2. Disagree
    3. Agree
    4. Strongly agree
