# Supplementary material for: Patients’ and professionals’ perspectives on implementation of opportunistic salpingectomy: a mixed-method study
Source: BMC Health Serv Res. 2021 Jul 25;21:736. doi: 10.1186/s12913-021-06767-9 (PMC8310584; doi:10.1186/s12913-021-06767-9)
Supplement: Supplementary file 5 — Additional file 5. Professionals’ questionnaire [file 12913_2021_6767_MOESM5_ESM.docx]

**ADDITIONAL FILE 5** Professionals’ questionnaire

Baseline characteristics

1. What is your gender?
   1. Female
   2. Male
2. What is your age?
3. What is your current position?
   1. Gynecologist
      1. How many years have you been working as a gynecologist?
      2. What/Which special interest(s) do you have?
4. Benign and/or endoscopy
5. Oncology
6. Urogynecology
7. Other
   1. Gynecological resident
      1. What year of training are you currently in?
      2. Which special interest have you chosen?
8. Benign and/or endoscopy
9. Oncology
10. Urogynecology
11. Other
12. Where are you currently working?
    1. In an academic hospital
    2. In a teaching hospital
    3. In a non-teaching hospital

Influencing factors at health care professional level

1. I am not familiar with OS
   1. Strongly disagree
   2. Disagree
   3. Agree
   4. Strongly agree
2. I am unaware of the evidence about OS
   1. Strongly disagree
   2. Disagree
   3. Agree
   4. Strongly agree
3. I do not know the benefits of performing OS
   1. Strongly disagree
   2. Disagree
   3. Agree
   4. Strongly agree
4. I have insufficient surgery skills to perform OS laparoscopic/laparotomic
   1. Strongly disagree
   2. Disagree
   3. Agree
   4. Strongly agree
5. I have insufficient surgery skills to perform OS vaginally
   1. Strongly disagree
   2. Disagree
   3. Agree
   4. Strongly agree
6. I am not convinced of the evidence about OS
   1. Strongly disagree
   2. Disagree
   3. Agree
   4. Strongly agree
7. During consultation I often forget to counsel on OS
   1. Strongly disagree
   2. Disagree
   3. Agree
   4. Strongly agree
8. I experience time stress during consultation through additional counselling on OS
   1. Strongly disagree
   2. Disagree
   3. Agree
   4. Strongly agree
9. I need additional counselling material about OS
   1. Strongly disagree
   2. Disagree
   3. Agree
   4. Strongly agree
10. I need a decision aid about OS as additional procedure during abdominal surgery
    1. Strongly disagree
    2. Disagree
    3. Agree
    4. Strongly agree
11. I need a decision aid about various sterilization methods including OS
    1. Strongly disagree
    2. Disagree
    3. Agree
    4. Strongly agree
12. I would counsel more if there is more evidence about the effectiveness of OS
    1. Strongly disagree
    2. Disagree
    3. Agree
    4. Strongly agree
13. I would counsel more if there is more evidence about the long term effects of OS
    1. Strongly disagree
    2. Disagree
    3. Agree
    4. Strongly agree
14. I would counsel more often on OS if I can use counselling material
    1. Strongly disagree
    2. Disagree
    3. Agree
    4. Strongly agree

Influencing factors at innovation (OS) level

1. Performing OS laparoscopic/laparotomic is successful in most cases
   1. Strongly disagree
   2. Disagree
   3. Agree
   4. Strongly agree
2. Performing OS vaginal is successful in most cases
   1. Strongly disagree
   2. Disagree
   3. Agree
   4. Strongly agree
3. OS can not be performed in all cases without additional risk
   1. Strongly disagree
   2. Disagree
   3. Agree
   4. Strongly agree
4. OS does not affect the size of laparoscopic/laparotomic surgery
   1. Strongly disagree
   2. Disagree
   3. Agree
   4. Strongly agree
5. OS does not affect the size of vaginal surgery
   1. Strongly disagree
   2. Disagree
   3. Agree
   4. Strongly agree
6. OS does not affect the size of sterilization
   1. Strongly disagree
   2. Disagree
   3. Agree
   4. Strongly agree
7. The evidence of long term risks and effects of OS are insufficient
   1. Strongly disagree
   2. Disagree
   3. Agree
   4. Strongly agree
8. The limits of the eligible population for OS are unclear
   1. Strongly disagree
   2. Disagree
   3. Agree
   4. Strongly agree

Influencing factors at patient level

1. The decision whether or not to undergo OS is very difficult for patients
   1. Strongly disagree
   2. Disagree
   3. Agree
   4. Strongly agree
2. Patients often request for OS themselves
   1. Strongly disagree
   2. Disagree
   3. Agree
   4. Strongly agree
3. Many patients have fear of earlier onset of menopause due to OS
   1. Strongly disagree
   2. Disagree
   3. Agree
   4. Strongly agree
4. Many patients prefer not to be at risk on earlier onset of menopause due to OS
   1. Strongly disagree
   2. Disagree
   3. Agree
   4. Strongly agree
5. Many patients prefer not to remove healthy organs if this is not necessary
   1. Strongly disagree
   2. Disagree
   3. Agree
   4. Strongly agree
6. Patients often have no knowledge of the disadvantages of OS
   1. Strongly disagree
   2. Disagree
   3. Agree
   4. Strongly agree
7. Patients often do not worry if OS can not be performed during surgery
   1. Strongly disagree
   2. Disagree
   3. Agree
   4. Strongly agree
8. Patients often have no insight into the size of the surgery
   1. Strongly disagree
   2. Disagree
   3. Agree
   4. Strongly agree
9. Patients often do not know the difference between the ovaries and fallopian tubes
   1. Strongly disagree
   2. Disagree
   3. Agree
   4. Strongly agree

Influencing factors at organizational, economic and political level

1. No extra instruments are necessary for performing OS
   1. Strongly disagree
   2. Disagree
   3. Agree
   4. Strongly agree
2. I need more time for counselling on OS, which increases my consultation time
   1. Strongly disagree
   2. Disagree
   3. Agree
   4. Strongly agree
3. I need more surgery time due to OS
   1. Strongly disagree
   2. Disagree
   3. Agree
   4. Strongly agree
4. An additional telephone consultation is necessary to give the patient more decision time
   1. Strongly disagree
   2. Disagree
   3. Agree
   4. Strongly agree
5. All fallopian tubes should be submitted for pathological analysis
   1. Strongly disagree
   2. Disagree
   3. Agree
   4. Strongly agree
6. I do not know how to invoice OS
   1. Strongly disagree
   2. Disagree
   3. Agree
   4. Strongly agree
7. I do not know which diagnose treatment combination (DBC) contains OS
   1. Strongly disagree
   2. Disagree
   3. Agree
   4. Strongly agree

Influencing factors at social level

1. Consensus on OS within the gynecology departments would advance the implementation
   1. Strongly disagree
   2. Disagree
   3. Agree
   4. Strongly agree
2. National consensus is necessary to ensure complete implementation
   1. Strongly disagree
   2. Disagree
   3. Agree
   4. Strongly agree
3. Including OS in the guidelines of NVOG would advance the implementation
   1. Strongly disagree
   2. Disagree
   3. Agree
   4. Strongly agree
4. Including OS in the guidelines of NHG would advance the implementation
   1. Strongly disagree
   2. Disagree
   3. Agree
   4. Strongly agree
